# Spontaneous Perinephric Hemorrhage Associated with Urolithiasis in Pregnancy

**DOI:** 10.1100/tsw.2004.69

**Published:** 2004-06-07

**Authors:** Leah P. McMann, Andrew C. Peterson, Sunil A. Ahuja

**Affiliations:** Department of Surgery, Urology Service Madigan Army Medical Center, Tacoma, WA 98431, USA

## Abstract

Spontaneous perinephric hematoma in the absence of anticoagulation, arteritis, or trauma is uncommon. We report the case of a postpartum patient with nephrolithiasis who initially presented to the obstetric service with a spontaneous perinephric hemorrhage.

## CASE REPORT

A 26-year-old G4P3 Asian female presented to the obstetric service at 38 weeks with severe unremitting low back pain and a known history of nephrolithiasis. A limited intravenous pyelogram (IVP) was performed, which showed a filling defect consistent with a nonobstructing right distal ureteric stone (Figure 1). She was made pain-free and labor was augmented the following day, complicated by a drop in her hematocrit from 33.1 % prior to delivery to 27.6 % on postpartum day 1. She was discharged from the hospital and presented five days later with headache, nausea, and vomiting. On physical exam, she was afebrile with a blood pressure of 164/92 and pulse of 61. She had right upper quadrant (RUQ) abdominal tenderness with no palpable masses. Liver transaminases were elevated. Bilirubin, platelet count, coagulation studies, and urinalysis were normal. A RUQ abdominal ultrasound showed a right perinephric fluid collection and computerized tomography (CT) scan of the abdomen showed a 9 cm dense lateral fluid collection contained within Gerotaís fascia and without extravasation of contrast (Figures 2a and b). A right ureteral stent was placed with subsequent spontaneous passage of the stone. Renal magnetic resonance imaging (MRI) and renal angiography revealed no evidence of neoplastic or vascular abnormalities.

## DISCUSSION

Spontaneous perinephric hematomas are uncommon. Renal tumors, vasculopathies, infectious disease, nephritis, and blood dyscrasias are the most commonly reported etiologies.[1-4] Ten cases of renal hemorrhage associated with parturition have been reported.[5-10] In these cases, the etiology of the bleeding was obscure, with no detectable pathology to account for the sudden onset of hemorrhage.[9] We report the first case of a spontaneous perinephric hematoma associated with urolithiasis in pregnancy. As shown by

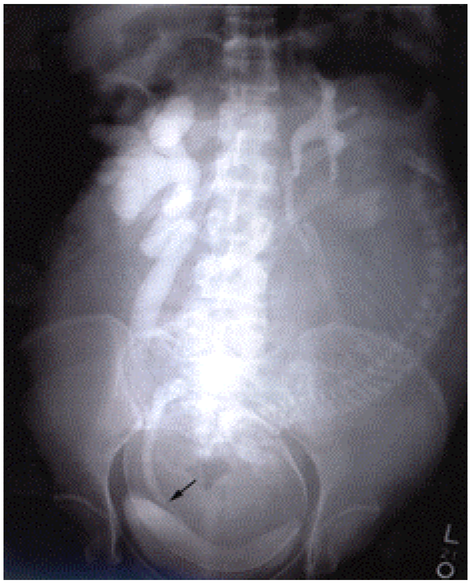

**FIGURE 1**. Intravenous pyelogram at term showing a filling defect at the right ureterovesical junction (arrow), consistent with the distal ureteral stone.

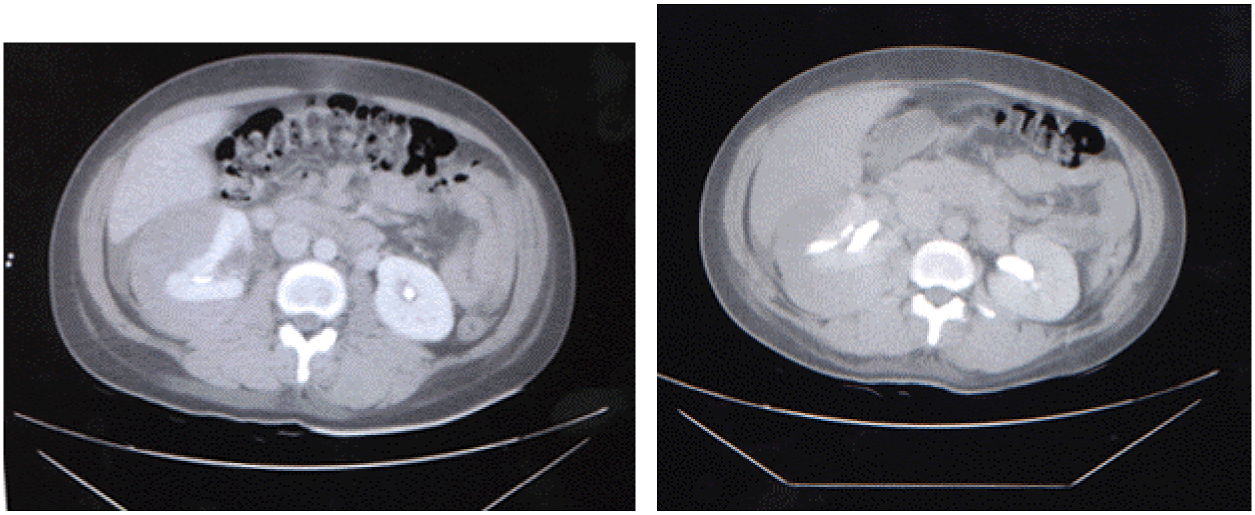

**FIGURE 2a and 2b**. Contrast enhanced computerized tomography scan showing a right perinephric hematoma within Gerota's fascia and without contrast extravasation.

her negative MRI and angiography, the patient had none of the previously reported risk factors for spontaneous hemorrhage, to include tumors, vasculopathies, infections, and blood dyscrasias. Nephrolithiasis in pregnancy should be considered a risk factor for spontaneous perinephric hematoma, along with the aforementioned disorders.
